# Link between emotional and external eating behaviors, peripheral neuropeptide Y, and β-hydroxybutyrate in participants with obesity on 12-week ketogenic diet

**DOI:** 10.1177/02601060231154464

**Published:** 2023-02-03

**Authors:** Maša Černelič-Bizjak, Saša Kenig, Ana Petelin, Zala Jenko-Pražnikar, Nina Mohorko

**Affiliations:** Faculty of Health Sciences, 68960 University of Primorska, Izola, Slovenia

**Keywords:** Emotional eating, external eating, stress-related obesity, peripheral neuropeptide y, β-hydroxybutyrate, ketogenic diet

## Abstract

**Objective:** Understanding the impact of stress on emotional and external eating behaviors and the psychological and the associated metabolic factors can help in designing subsequent interventions to protect health. In particular, psychological trait-like construct related to eating has been shown to be an important target for intervention. **Methods and measures:** This study aimed to investigate the biochemical variables associated with a decrease in emotional and external eating behaviors due to 12-week ketogenic diet (12KD) in 35 adult participants (12 males) with obesity. **Results:** Absolute changes in emotional and external eating were independent of changes in body mass, nutritional intake, and Δ cortisol, but were predicted with increases in serum β-hydroxybutyrate (BHB) and decreases in serum peripheral neuropeptide Y (pNPY) (all *p’*s < 0.050). Decrease in pNPY was also associated with an increase in BHB but was independent of anthropometrical changes, Δ fasting glucose, and Δ insulin. **Conclusion:** The reductions in emotional and external eating behaviors in participants with obesity were uniquely predicted by an increase in BHB and a decrease in pNPY after 12KD. In ketosis, emotional and external eating dropped independently of body mass change. Change in pNPY predicted changes in emotional and external eating. The role of BHB in modulating eating behavior should be further explored.

## Introduction

Energy intake (EI) which may be influenced by distinct behavioral and metabolic components is a consequence of eating behavior (Hopkins and Blundell, 2016; Vainik et al., 2019). Although eating and stress and its contributions to obesity are not clearly delineated, it seems that eating behavior can be stress-induced (Torres and Nowson, 2007). Stress affects the type and amount of food people choose, and many people report consuming more energy and shifting their food choices to a greater proportion of energy-dense highly palatable food in response to stress (Ulrich-Lai et al., 2015). Eating behavior is the result of numerous complex regulatory mechanisms involving both homeostatic and non-homeostatic processes that are intertwined and can manifest as eating in the absence of energy or metabolic deficits—overeating (Liu and Kanoski, 2018). Based on emotional (irritability, anxiety or stress) and external (smell or sight of attractive food) cues, we can distinguish two eating behaviors, emotional and external (van Strien, 2018; van Strien et al., 2009). Literature reports associations of emotional eating behavior with the consumption of energy-dense snacks and sweet and fatty foods (Camilleri et al., 2014) and unhealthy weight gain with abdominal obesity (Konttinen et al., 2019). These increase the risk of developing metabolic syndrome (Agodi et al., 2018), eating disorders, and binge eating episodes (Stice et al., 2002). Reducing this psychological trait-like construct related to eating may therefore be an important treatment target for obesity and other health outcomes.

It seems that stress might influence physiologic mechanisms related to obesity too. Peripherally, stress response is mediated by two systems, sympatho-adrenomedullary system (SAS) and hypothalamic–pituitary–adrenal axis (HPA) with cortisol as the final hormone in human. Although the best known SAS mediators are adrenalin and noradrenalin (NA), in sympathetic activation, neuropeptide Y (NPY) is co-transmitted with NA (Ekblad et al., 1984; Hirsch and Zukowska, 2012). Peripheral NPY (pNPY) acts directly on fat tissue and mediates stress-induced obesity and metabolic syndrome (Kuo et al., 2007) and is important in immune (Wheway et al., 2005) and cardiovascular function (Pedrazzini et al., 1993; Zhang et al., 2012). Chronically elevated pNPY was observed in circulation in patients with obesity compared to subjects with healthy body mass (Zhang et al., 2011).

Stress-related obesogenic response is commonly an overlooked target for obesity management, but important to increase its efficiency (Tomiyama, 2019). Ketogenic diet (KD), an efficient tool for weight loss, was associated with reductions in negative emotions (McClernon et al., 2007), may have antidepressant properties (Murphy et al., 2004), positive effects on mood (Yancy et al., 2009). Recent studies (Carmen et al., 2020; Dalai et al., 2020) support the use of a low-carbohydrate KD as a therapeutic approach for ultra-processed food addiction and binge-eating symptoms. The most recent pilot study (Rostanzo et al., 2021) assessed the use of a KD in women with addictive eating disorders who want to lose weight. This study highlighted the potential therapeutic role of KD in treating addiction to high-calorie, extremely processed, and high-glycemic foods. Currently, there are limited data on the potential effects of KD on emotion, and results from human studies are limited. Existing studies (McClernon et al., 2007) examined the effects of diet on mood, hunger, and other related symptoms in obese patients. They found that patients on KD had decreased hunger and food cravings compared to patients on low-fat diets, with fewer negative effects. KD is showing potential as a strategy for other behavioral addictions, too. Animal studies have shown that a KD reduces alcohol consumption in adult male mice (Blanco-Gandía et al., 2021) and decreases behavioral responses to cocaine in young adult male and female rats (Martinez et al., 2019). Recent evidence shows efficacy of KD in the treatment of various conditions such as obesity (Tragni et al., 2021; Verde et al., 2023), dislipidemia, and cardiovascular risk factors (Tragni et al., 2021), fatty liver disease (Luukkonen et al., 2020), enhancement of quality of life, physical performance, body composition, and metabolic health in women with breast cancer (Kämmerer et al., 2021), and in adult patients with epilepsy (Green et al., 2020). Nutritional ketosis has a significant impact on the metabolic profile by lowering and stabilizing insulin levels and therefore increasing the ability to use fat (both external from food and internal from adipose stores) and suppressing fatty acid synthesis (Gershuni et al., 2018; Newman and Verdin, 2014). This is opposite to the insulin-dictated glucose-dependent state that increases the fatty acid synthesis and inhibits fat mobilization from adipose stores (Gershuni et al., 2018; Newman and Verdin, 2014). It has been suggested that ketone bodies, especially β-hydroxybutyrate (BHB), might act as signaling metabolites (Newman and Verdin, 2014). We observed a decrease in emotional and external eating behaviors after 12-week ketogenic diet (12KD) in participants with obesity (Mohorko et al., 2019), but a comprehensive determination of such diet and its relationship to eating behaviors is still largely unexplored. Therefore, our aim in the present study was to identify the possible metabolic parameters through which the KD influenced eating behavior by exploring the relations between absolute changes in BHB levels in serum, health characteristics that significantly changed in nutritional ketosis, pNPY and cortisol, measured in serum, and emotional and external eating after 12KD.

## Methods

### Study design, participants, and intervention

The study design and dietary intervention of 12KD were concisely described previously (Mohorko et al., 2019). To sum up, this single-arm intervention study with repeated measurements was conducted at the University of Primorska, Slovenia. Subjects with obesity were assigned to consume KD for 12 weeks and were requested to maintain their habitual physical activity level. The protocol was approved by the Republic of Slovenia Ministry of Health National Medical Ethics Committee (0120-100/2017) and was registered in ClinicalTrials.gov (NCT03338452). All procedures were performed in agreement with the ethical guidelines on biomedical research on human subjects of the Declaration of Helsinki. We calculated a minimum sample size of 31 participants, based on the SD of the change in the insulin level 1.0 μU/mL, type I error probability α = 0.05, effect size of 0.500, and power 1−β = 0.8. Inclusion criteria were willingness to eat the foods prescribed in the study, BMI higher than 30 kg/m^2^, stable weight 3 months prior to intervention start, absence of cardiovascular, endocrine, acute or chronic inflammatory diseases, and not taking medications for lipid metabolism or psychiatric disorders. A physician confirmed suitable health conditions of the participants through a complete review of medical history and a physical examination.

Of 38 eligible participants (out of 45 volunteers), 35 adults, aged between 30 and 45 years (37 ± 7 years; 23 females) completed the 12-week intervention study and their data was further investigated in the present study. Participants had an average BMI of 36.1 ± 5.6 kg/m^2^ at baseline, and there were no significant differences in age or BMI between the sexes. In the first 2 weeks, participants were instructed to follow a low-energy KD (5–10% EI from carbohydrates, at least 75% EI from fat and 20% EI from protein) with deliberately low EI (1200–1500 kcal); however, after week 2, they were free to ad libitum EI with the prescribed macronutrient composition of the diet with low carbohydrate intake and high-fat intake (as in the first 2 weeks: 5–10% EI from carbohydrates, at least 75% EI from fat and 20% EI from protein). EI and nutritional intake were assessed with a 3-day food record. Despite the ad libitum EI, the participants’ EI significantly decreased; significant changes were observed also in macronutrient intake and ketosis, confirmed by significantly increased serum BHB from week 1 (*p* < 0.01) (Mohorko et al., 2019). The participants underwent a series of measurements and consultations at baseline (prior to the start of the intervention), and at weeks 4 and 12 of 12KD.

### Eating behavior and body satisfaction evaluation

The Dutch Eating Behavior Questionnaire (DEBQ) translated to Slovenian was employed at baseline and post 12KD to assess emotional, external, and restrained eating behavior (van Strien et al., 1986). DEBQ has 33-items with 5-choice answers (ranging from “never” to “very often”), divided into 3 scales: 13 questions on “emotional eating,” 10 on “external eating,” and 10 on “restrained eating.” The author of the DEBQ reports high internal consistency reliabilities (alphas) across weight category groups. Body satisfaction was assessed with body dissatisfaction subscale from the Eating Disorders Inventory-2 (EDI-2) (Garner, 1991).

### Biochemical and hormonal changes evaluation

Biochemical and hormonal serum measurements were described previously (Mohorko et al., 2019). In brief, venous blood samples for biochemical and hormonal determinations were collected at baseline and after 12KD in the morning between 7 am and 10 am after overnight fasting. Serum was immediately separated, frozen and stored at −80°C until subsequent analysis. Serum concentrations of BHB, pNPY, and cortisol were determined using human ELISA Kits (BHB (Cayman Chemical Company, USA), NPY (EMD Millipore Corporation, USA) and cortisol (IBL international GMBH, Germany)). Serum concentrations of glucose and C-reactive protein (CRP) were measured using Cobass reagents and performed on Cobass c111 analyzer (Roche). Serum insulin concentrations were measured using Abbott reagents and performed on 2000 iSR analyzer (Abbott Architect).

### Statistical analysis

Data were analyzed using IBM SPSS Statistics version 23.0. The hypotheses were specified before the data were collected and the analytic plan was pre-specified.

All variables were tested for normal distribution. Student's paired *t*-tests were used to investigate the effect of intervention on eating behavior.

Absolute changes (Δ) from baseline and week 12 were calculated for eating behaviors, biological variables, and nutrient composition and were used for associational analysis.

Hierarchical multiple regressions were used to determine the extent to which the studied variables can be viewed as predictors of the emotional and external eating behaviors. Thus, two hierarchical multiple regression analyses were performed to examine the effects of absolute changes in fat mass, dietary intake, and five biological markers on the changes in level of eating behaviors. Post hoc, observed power for the multiple regression analysis was calculated, given the observed probability level, the number of predictors, the observed R^2^, and the sample size.

Pearson's correlation analyses were performed to examine the associations between Δ pNPY and changes in several serum indicators of hormones and metabolites. Statistical significance was set at *p* < 0.05.

Relations among Δ BHB, Δ pNPY, and absolute change in eating behaviors were represented with bubble charts. Δ BHB is represented on *x*-axis, Δ pNPY on *y*-axis, while color gradient represents changes in values of eating behaviors.

## Results

### 12KD induced significant changes in participants

The timeline of changes due to 12KD has been published (Mohorko et al., 2019). Absolute changes in parameters of interest to the present analysis are reported in Table 1.

**Table 1.** Absolute changes in participants’ biochemical and hormonal characteristics after 12-week ketogenic diet (12KD).

Parameter	12-week KD
Δ energy intake (EI) (kcal/day)	−794 ± 503
Δ fat mass (%)	−10.5 ± 2.2
Δ carbohydrate intake (g/day)	−131 ± 36
Δ fatty acid intake (g/day)	−14 ± 26
Δ protein intake (g/day)	−31 ± 22
Δ BHB (mmol/L)	0.51 ± 0.73
Δ CRP (mg/L)	−0.38 ± 1.23
Δ pNPY (pg/mL)	0.17 ± 4.18
Δ fasting insulin (mU/L)	−2.89 ± 2.45
Δ cortisol (µg/dL)	0.22 ± 0.73
Δ body dissatisfaction	−9 ± 7

BHB: β-hydroxybutyrate; CRP: C-reactive protein; pNPY: peripheral neuropeptide Y; Δ: absolute change from baseline. The reported data are expressed as means ± SD.

### Significant improvement in emotional and external eating behaviors

**Figure 1.**
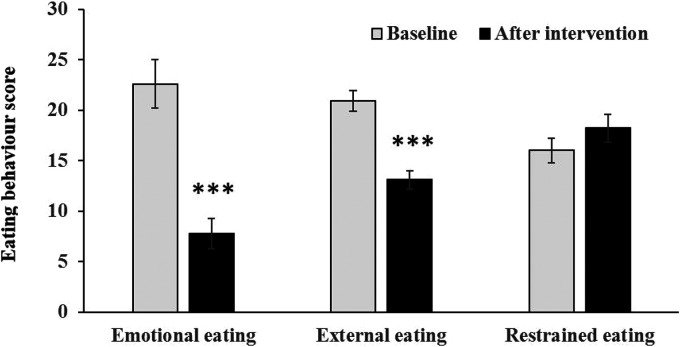
Eating behaviors on baseline and after 12-week ketogenic diet (12KD) (***p < 0.001).

A significant improvement in eating behaviors was observed (Mohorko et al., 2019): there were significant reductions in emotional and external eating behaviors (both *p* < 0.001) while restrained eating did not change significantly (*p* = 0.106) (Figure 1).

### Δ BHB and δ NPY predicted changes in eating behaviors

Multiple linear regression models for emotional and external eating that took into account general participants’ characteristics, participants’ changes in nutritional intake, changes in biochemical parameters, and changes in body satisfaction identified ΔBHB and ΔpNPY as predictors for changes in emotional and external eating dimensions are presented in Table 2. The post hoc observed power of the analysis, given the observed probability level of 0.05, the number of predictors (12), the observed R^2^ (0.657 for emotional eating and 0.597 for external eating), and the sample size of 31 was 0.99 for emotional eating and 0.96 for external eating.

**Table 2.** Predictors of emotional and external eating behaviors.

	Eating behavior								
	Δ emotional eating					Δ external eating			
Predictor	*r*	Δ*R*^2^	*β*	*F*		*r*	Δ*R*^2^	*β*	*F*
Step 1		0.084		0.786			0.127		2.855
Sex	−0.12		−0.13			−0.25		−0.26	
Age (years)	0.10		0.09			−0.24		−0.22	
Δ fat mass (kg)	0.17		0.15			0.17		0.16	
Step 2		0.129		1.623			0.175		1.867
Δ energy intake (EI) (kcal/day)	0.14		0.13			0.30		0.29	
Δ CHO intake (g/day)	0.11		0.09			0.10		0.09	
Δ FA intake (g/day)	0.07		0.08			0.25		0.21	
Δ protein intake (g/day)	0.15		0.17			0.31		0.31	
Step 3		0.320*		6.524			0.250*		4.391
Δ BHB (mmol/L)	−0.43*		−0.38*			−0.44*		**−0.41**	
Δ CRP (mg/L)	0.28		0.23			0.18		0.17	
Δ fasting insulin (mU/L)	0.33		0.29			0.35*		0.33	
Δ cortisol (μg/dL)	0.29		0.31			0.20		0.21	
Δ pNPY (pg/mL)	0.43*		0.40*			0.37*		0.38*	
Step 4		0.131		2.078			0.045		1.315
Δ body dissatisfaction	0.35		0.28			0.25		0.23	
Total *R*^2^		0.657					0.597		
*N*	35					35			

Post hoc observed power: 0.99 (emotional eating) and 0.96 (external eating). Significant predictors, that is, Δ BHB and Δ pNPY; are marked in bold.

*p < 0.050.

BHB: β-hydroxybutyrate; CHO: carbohydrates; CRP: C-reactive protein; FA: fatty acids; pNPY: peripheral neuropeptide Y; Δ: absolute change.

### Factors associated with δ pNPY

As Δ pNPY and Δ BHB were the only predictors of emotional and external eating in our multiple linear regression models, we performed Pearson's correlations analyses to investigate possible factors related to Δ pNPY and changes in other parameters observed in ketosis. Significant negative correlations between Δ pNPY and Δ BHB and pNPY and Δ CRP were observed (Table 3).

**Table 3.** Pearson's correlation analyses between δ pNPY and absolute changes from baseline in other parameters due to 12-week ketogenic diet (12KD).

Independent variable	Δ pNPY	
	r	*P*
Age (years)	0.129	0.505
Δ EI (kcal/day)	0.265	0.182
Δ fat mass (%)	−0.251	0.206
Δ BHB (mmol/L)	**−0.423**	0.023*
Δ fasting insulin (mU/L)	0.268	0.311
Δ CRP (mg/L)	**−0.311**	0.034*
Δ cortisol (μg/dL)	−0.263	0.079

*p < 0.050.

BHB, β-hydroxybutyrate; CRP, C-reactive protein; EI, energy intake; pNPY, peripheral neuropeptide Y; Δ: absolute change.

**Figure 2.**
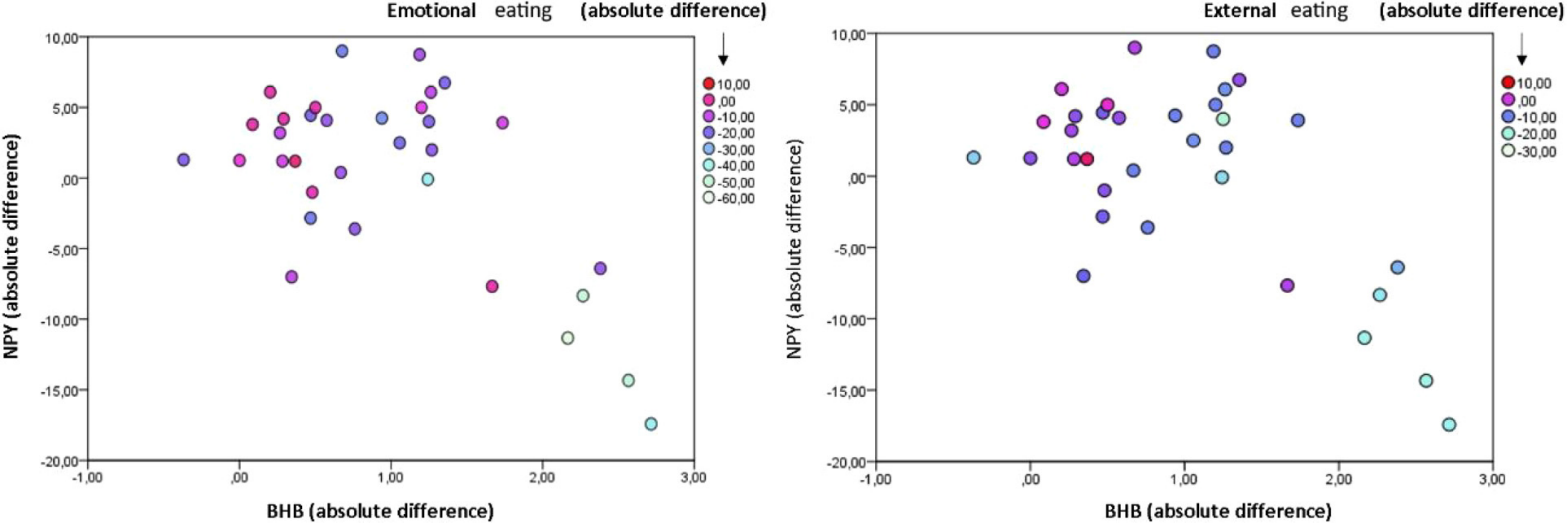
Δ BHB in relation to δ pNPY and absolute change in eating behaviors. (a) Negative association between δ BHB and δ pNPY and changes in emotional eating. (b) Negative association between Δ BHB and Δ pNPY and changes in external eating. Color gradient represents changes in values of eating behaviors.

BHB, β-hydroxybutyrate; pNPY, peripheral neuropeptide.

There was a three-way relation between changes in eating behaviors, ΔBHB and ΔpNPY (Figure 2a). Reductions in eating behaviors were associated with an increase in BHB and a decrease in pNPY was also associated with increase in BHB (Figure 2).

## Discussion

In present study, the relationship of change in BHB, pNPY and emotional and external eating behaviors during nutritional ketosis are described. The reduction in the two eating behaviors among asymptomatic middle-aged adults with obesity after 12KD was uniquely predicted by an increase in serum BHB and a decrease in serum pNPY. Interestingly, the changes in obesity indicators, such as substantially decreased body and fat mass and low carbohydrate intake or high-fat intake, did not correlate nor predict either emotional or external eating behaviors. The rates of change in BMI and the eating behavior are not always correlated (Vainik et al., 2019). Food cravings, which contribute to overeating, have been previously reported to decrease progressively with EI reduction (Martin et al., 2006). Spontaneous reduction in EI when carbohydrate intake was 5–10% of EI was reported (Johnstone et al., 2008; Mohorko et al., 2019). To obtain the effect of food intake inhibition, it seems to be important that energy restriction is associated with high-fat intake. Previous studies show that KD with a high percentage of EI from fat reduced appetite (Choi et al., 2018) whereas low energy KD with a high percentage of EI from carbohydrates reduced feeling of hunger only postprandially when the subjects were ketotic, whereas fasting feelings of hunger significantly increased despite ketosis (Lyngstad et al., 2019). Studies in laboratory animals showed that high-fat-associated ketosis blunted other nutrient sensing in the hypothalamus and delayed further food intake (Le Foll et al., 2014). Studies in human subjects did not report changes in ghrelin levels in high-fat KD (Castro et al., 2018; Lyngstad et al., 2019; Mohorko et al., 2019). Further, the reductions in emotional and external eating were predicted by increase in BHB, independently of sex, age, and Δ fat mass which might point to an active role of BHB as a signaling metabolite in regulating food intake, which has to be explored.

Cortisol is reported to be an important mediator of appetite and of stress-induced eating (Michels, 2019), but Δ cortisol neither correlated with nor predicted the eating behaviors in our study. Transient increase in cortisol was not accompanied by increased food intake, on the contrary, participants reported less hunger and spontaneously decreased EI (Mohorko et al., 2019). We did not find any similar data from other human studies, but a similar report came from a study with high-fat and sugar diet-fed mice subjected to stress where cortisol was not associated with increased fat mass (Kuo et al., 2008). In nonobese rats under stress, nutritional ketosis did not affect HPA response to acute stressor nor did it affect the stress-related loss of body weight (Brownlow et al., 2017) which is in line with our observation of no associations between Δ BHB and Δ cortisol and the independence of observed changes in eating behaviors and changes in body composition. As reported previously, we did not observe any significant changes in mood (depression or anxiety) during the intervention and all participants were within the normal nonclinical range for each psychometric measure before and after 12KD (Mohorko et al., 2019). Body image satisfaction significantly improved, but this change did not correlate with changes in emotional and external eating.

A strong predictor of changes in emotional and external eating behaviors was Δ pNPY. The latter has consistently been found to increase in obesity (Zhang et al., 2011), adipocytes were found to secrete pNPY (Kos et al., 2007) and it was even proposed that pNPY might be a biomarker of obesity (Zhang et al., 2014). pNPY is also the product of SAS, where it is co-transmitted with NA (Ekblad et al., 1984; Morris et al., 1986) and is strongly implicated in the stress-related visceral fat mass expansion (Hirsch and Zukowska, 2012). Nevertheless, we did not find any associations between Δ fat mass and Δ pNPY, no any associations between Δ insulin and Δ pNPY. Δ pNPY was strongly negatively associated with Δ BHB, showing an important role of nutritional ketosis in diminishing pNPY levels; and since mood parameters in our study remained unchanged (Mohorko et al., 2019), this calls for a need to investigate the possible role of nutritional ketosis in attenuating pNPY. This is further supported by the fact that chronic stress needs to be in combination with high-fat and sugar comfort food diet to increase sympathetic NPY production with no concomitant increase in NA production (Kuo et al., 2008).

We found a three-way relation between an increase in BHB, a decrease in pNPY, and reduction in eating behaviors, which points to a possible link between them. For a more detailed interpretation of this result, it would be important to know how our peripherally detected pNPY concentrations reflect concentrations or actions of central NPY, which is strongly implicated in both, food intake regulation and response to stress (Comeras et al., 2019; Ip et al., 2019), and it also influences sympathetic innervation (Chao et al., 2011). NPY has been shown to cross the blood–brain barrier in a mouse model (Kastin and Akerstrom, 1999), but a study comparing central (cerebrospinal fluid) and peripheral (plasma) NPY concentration in humans found no correlation between the two (Baker et al., 2013). If pNPY and central NPY are independent, BHB could be a mediator between central and peripheral responses.

The main limitations of our study are that we did not have a control group and that the number of participants was too small to perform statistical analysis separately for each sex and compare them to assess any possible sex differences. Since literature reports higher scores of emotional eating behavior in women (Larsen et al., 2006), further research should be designed in order to take in consideration possible sex differences.

## Conclusions

Reductions in emotional and external eating behaviors in participants with obesity were predicted by an increase in BHB and a decrease in pNPY after 12KD. Moreover, BHB negatively correlated with pNPY levels. Observed associations were independent of obesity indicators, such as fat mass and lower carbohydrate intake or increased fat intake and other stress-related parameters such as cortisol, which points to a role of BHB in regulating observed psychological and physiological responses. The role of high-fat KDs that induce nutritional ketosis and BHB as a candidate signaling molecule in dimensions related to eating, and pNPY, which seem to be correlated, has to be further explored in controlled studies and bigger sample for their potential positive impact on eating behavior and stress in obese.
